# Characteristics of daily foraging activity of Camponotus japonicus via time series analysis

**DOI:** 10.1371/journal.pone.0293455

**Published:** 2023-11-16

**Authors:** Hiromichi Goko, Osamu Yamanaka, Masashi Shiraishi, Hiraku Nishimori

**Affiliations:** 1 Frontier Research Center, Toyota Motor Corporation, Toyota, Aichi, Japan; 2 Meiji Institute for Advanced Study of Mathematical Sciences, Meiji University, Nakano, Tokyo Japan; University of the Philippines Diliman, PHILIPPINES

## Abstract

Social insects often share tasks among individuals. In this study, we analyzed the foraging activity of ants (*Camponotus japonicus*) and recorded the daily passage event counts of individual workers between a nest chamber and a foraging arena in five monodomous colonies. We proposed two hypotheses on the time series of foraging frequency by individual worker ants as follows: (i) for the time series of foraging frequency by individual worker ants, the foraging frequency on a certain day could be expressed by the product of the foraging frequency on the previous day and the exponential of a random number. (ii) The random numbers are correlated between some pairs of worker ants. The results for the five tested ant colonies showed that the probability of total daily passage counts (the sum of an individual’s passage count) followed a log-normal distribution. The worker ants behaved differently in terms of active days and foraging frequency. However, for > 54% of the worker ants, the probability of the daily passage count was characterized by a log-normal distribution, and these worker ants performed > 72% of the tasks in each colony. Furthermore, for > 73% of the worker ants, the time development of the passage count was mathematically modeled; the logarithmic first difference between the passage counts on a certain day and those on the previous day was a random normal variable. These results support hypothesis (i). Additionally, the random numbers that were equivalent to the logarithmic first difference were correlated for some pairs of worker ants. These results support hypothesis (ii).

## Introduction

Worker ants share essential tasks to sustain their colony, such as caring for their broods, foraging for food, and protecting the colony from predators; the workload is too massive to be handled by an individual worker. The fact that the taxon has survived for 50 million years or longer implies that rational mechanisms of task allocation exist among workers, which aid in the sustenance of ant colonies [1]. The task allocation mechanisms of social insects have been explored theoretically [2] and experimentally [3, 4]. The colonies of social insects do not have leaders who supervise the workers [1]. Therefore, each worker chooses a task based on independent retrieval of external and internal information.

To obtain external information, they walk inside and outside of their nest and contact other workers through trophallaxis and antennation. Humidity conditions outside the nest regulate the foraging activity of the workers. The workers do not walk outside when humidity is low because it can cause dehydration and death [5]. Workers allocated with a specific task often communicate with other similarly specialized workers [6].

As for the internal information, the circadian rhythm is crucial in controlling the activity of individual workers, especially when they engage in brood care [7] and foraging [8, 9]. Although the circadian rhythm is an intra-individual trait, it acts as an external cue for other individuals during social interactions. In the monomorphic ant *Diacamma*, the individual workers display circadian rhythm regardless of age [7]. The interaction amongst the young workers entrained their circadian rhythm, which amongst the old workers did not result in any change. The presence of young workers in a mixed group entrains the collective circadian activity rhythm of the group.

Genetic information is another type of internal information that determines individual behavior. For example, *Pheidole* workers present phenotypically defined bimodal body sizes [10]. The workers with larger heads mainly guard the nest, but they also nurse their brood in the event of a reduction in the number of nursing workers. As a result of the complex information-sharing processes within and among individual workers, they spontaneously engage in tasks, making them appear as though the ants are assigned specific functions in the colony.

Task allocation is a macroscopic phenomenon accompanied by fluctuations, thus, the statistical properties of the counts and times of tasks performed by workers have been studied to characterize task allocation [11–13]. For example, statistical analysis of workload distribution of an entire colony revealed that the proportion of diligent and lazy workers was maintained after removing a part of the worker populations [14] and the shape of the workload distribution curve varied depending on the type of task [11]. These distribution data were obtained from a time series of activity. However, the time series of foraging activity has not yet been fully characterized because it requires an enormous amount of data for statistical validation. In terms of time-variation of statistical properties, the activity of individual workers is dynamically transient over time, with the statistical property being constant overall [15].

The probability distributions can be expressed by heavy-tailed distributions such as Ĺevy flight and log-normal [16–19]. Previous studies have also revealed the origin of such distributions [20–22]. For example, log-normal distribution is obtained under a mathematical premise [23]. These studies deepened the comprehensive understanding of animal behaviors and their commonality.

The foraging activity is evidently a vital task exhibited by ants. In this study, we recorded the foraging activities of the ant species, *Camponotus japonicus*. The foraging activity of *C. japonicus* has not been sufficiently analyzed from a statistical viewpoint using massive amounts of time series data. Our study was based on the following hypotheses:(i) the foraging frequency on a certain day could be expressed by the product of the foraging frequency on the previous day and a random number. (ii) The random numbers are correlated between pairs of worker ants, indicating that they collaborate. The first hypothesis (hypothesis (i)) is regarding the daily foraging frequency by individual worker ants. The random number corresponded to external and internal information of an ant and a colony. The second hypothesis (hypothesis (ii)) suggests that ants collaborate.

The two hypothesis ((i) and (ii)) are related in the following context: First, hypothesis (i) implies that the time series of the daily foraging frequency (DFF) of individual worker ants complies with the multiplicative stochastic process [24], which generates a log-normal distribution of the corresponding variable. To check whether this mathematical discussion was met in our study, we examined the log-normality of the distribution of DFF. Next, we explored the daily sum distribution of the foraging frequency of all ants in each colony and confirmed its log-normality. Notably, it has mathematically been recognized that the sum of different groups of random variables, each of which obeys a log-normal distribution, does not generally exhibit a log-normal distribution if the random variables in different groups are uncorrelated [25].

A logical suggestion obtained from the above two discussions, in conjunction with discussions from mathematical and experimental viewpoints, suggests a correlation of foraging behavior between different ants in each colony. To validate this suggestion, we analyzed the time-series correlation of daily foraging activities of different ants and confirmed its reliability.

## Materials and methods

### Materials and animal care [15]

Five colonies of *Camponotus japonicus* were collected from the Higashi-Hiroshima campus, Hiroshima University between June 2015, and June 2018. *Camponotus japonicus* is monogynous and polymorphic (the body size of an individual worker ranges from 7– to 12*mm*). The ant colonies A, B, and C, contained one queen each, whereas the two remaining colonies, D and E, were without a queen. Each colony had approximately 150 workers. The colonies were maintained in plastic cases patched with plaster to maintain humidity and wrapped in tape to prevent exposure to light.

### Experimental setup

**Fig 1:**
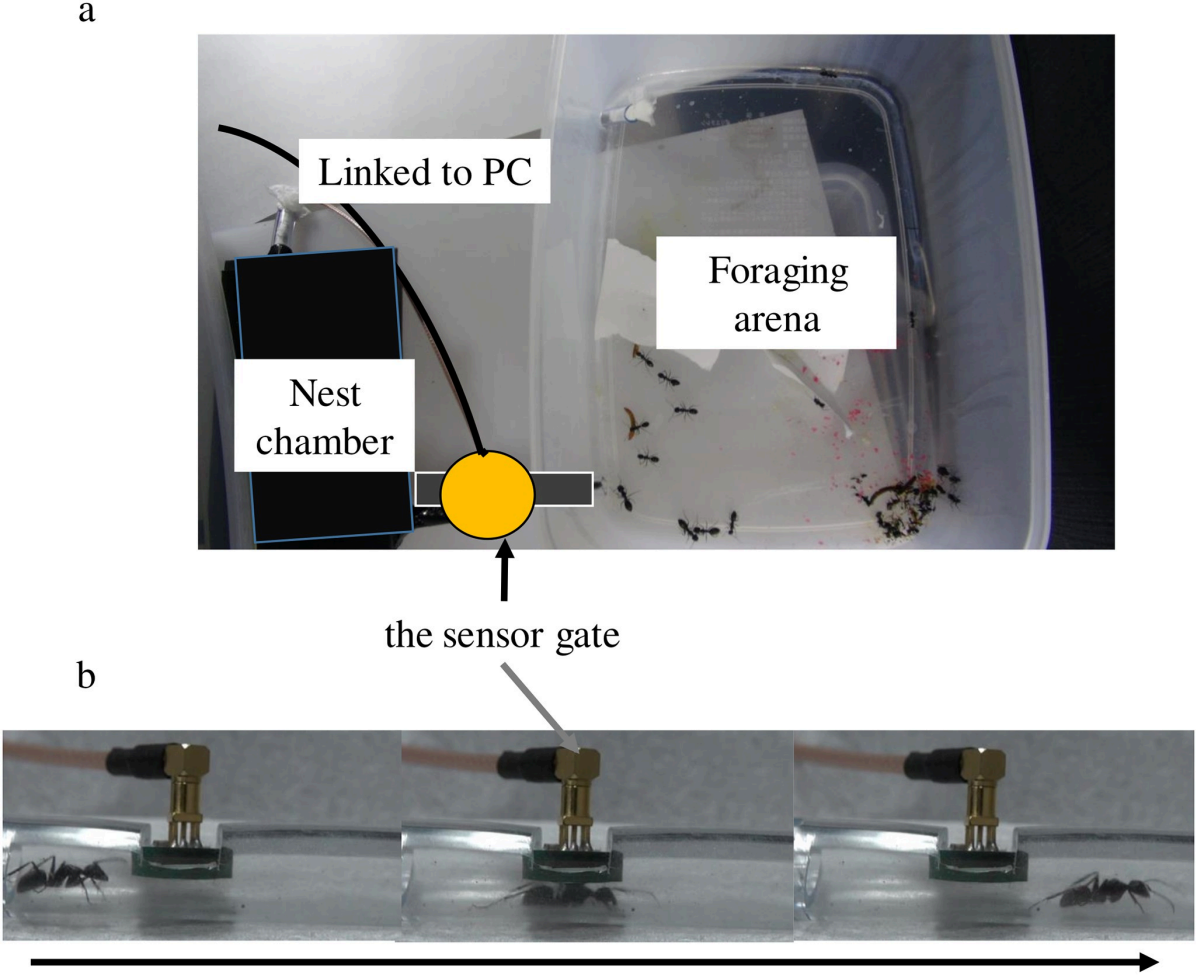
Experimental setups. Automatic system for measuring the foraging activity of *Camponotus japonicus* colonies using tiny radio frequency identification (RFID) tags. (a) Top view of the experimental setup. (b) Side view of the sensor gate and snapshots of a passage event of an ant moving from the nest chamber (left) to the foraging arena (right) in a time sequence.

All five monodomous colonies were maintained in an experimental setup consisting of a nest chamber and a foraging arena as shown in Fig 1a. The nest chamber and the foraging arena were connected by a rubber tube. The walls in the foraging arena were coated with Fluon to prevent workers from escaping. The experiment was performed in a room where a 12*h* light-dark daily cycle was maintained starting at 8 : 00 when the LED lights were switched on; the temperature was 25°C, and the humidity was higher than 50%. We added insect jelly continuously and mealworms once every 2 days in the foraging arena to maintain the foraging activity of the workers. Experiments were conducted from May 1, 2015, to August 18, 2015 (Colony A); from June 3, 2018, to October 1, 2018 (Colony B); from June 6, 2018, to October 1, 2018 (Colony C); and from June 26, 2018, to October 1, 2018 (Colonies D and E). All colonies were recorded on all days during their respective experimental periods using a radio frequency identifier (RFID) measuring system.

### RFID tag attachment to individual workers [15]

Radio frequency identification (RFIDs) (SK-Electronics CO., Ltd., Japan) with unique identification numbers were attached to the thoraxes of each worker ant but not the queen ants, using an acrylic resin glue (Kiyohara UVR) (KIYOHARA & Co., Ltd.) without exposure to CO_2_. The RFID tag weights were lower than those used in other studies [26–29]; therefore, the effect of the tags on worker behavior was considered negligible. After the RFID tags were attached to 10 workers, they were placed in a case and irradiated with ultraviolet light for 30 min to harden the glue. This process was repeated for all workers. Thereafter, they were released into the foraging arena before they returned to the nest chamber. Workers equipped with tags took almost one week to acclimatize to the new nest chamber and foraging arena. Considering the adjustment period, experiments were started 1 week after the workers entered the nest chamber. Newly emerged workers were not equipped with RFID tags. An RFID reader (hereafter referred to as the “sensor gate”) was attached to the inner, upper surface of the center of the rubber tube that connected the foraging arena and the nest chamber (Fig 1). The RFIDs of individual workers and corresponding time stamps were automatically recorded. The passage direction to and from the nest chamber and the foraging arena was not distinguishable by this system.

### Data selection

We collected data from worker ants whose main task was foraging. We excluded all other workers that foraged only a few times or once over a prolonged period because they were engaged in other main tasks besides foraging. The selected worker ants satisfied three conditions:

(I) The total number of passage events during its active period was at least 30.
(II) The active period was at least 10 days.
(III) An ant was active on at least half of the days during its active period.

The “active period” was defined as the set of days, from the first day to the last day, during which an ant made at least one passage event.

If a passage event was recorded on a certain day, we termed this day an “active day” and the ant an “active worker ant” of the day. For the time series analysis, we added 1 to the number of passage events in each day during the active period to avoid calculating log0.

**Fig 2:**
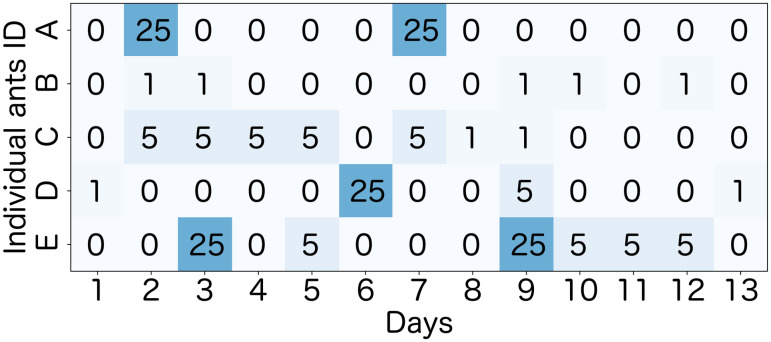
Virtual data of the foraging frequency of five ants in 13 days. Each number in the grid represents the foraging frequency of individual ants on a particular day. The shade of the background color indicates the intensity of foraging frequency. The active period, the active days, the number of passage events, and the satisfied condition for each virtual ant are shown in Table 1.

Fig 2 illustrates virtual data that consists of the foraging frequency of five ants in 13 days. In this case, only *Ant E* was selected as a worker ant because it met the above three conditions, (I), (II), and (III)(see Table 1).

**Table 1:** Active period, active days, the number of passage events, and satisfied condition for the five virtual ants in Fig 2.

Individual Ant ID	Active period	Active days	The number of passage events	Satisfied conditions
A	6	2	50	(I)
B	11	5	6	(II)
C	8	7	27	(III)
D	13	4	32	(I),(II)
E	10	6	70	(I),(II),(III)

### Statistical analyses

#### Static analysis

In the case of individual worker ants, hypotheses (i) could be expressed as follows.

$$\begin{array}{c} n_{i,t}=n_{i,t-1}\;\text{exp}\left(\epsilon_{i,t}\right). \end{array}$$ (1)

where *n*_*i*,*t*_ was defined as the count of passage events of the ant *i* on the day *t* and *ϵ*_*i*,*t*_ was an independent normal random variable. Note that *n*_*i*,*t*_ were hypothesized to be correlated for some pairs of ants (hypothesis (ii)).

Using simple mathematical discussion, we can recognize that, if Eq (1) holds and *ϵ*_*i*,*t*_ is an independent normal random variable, static distribution of passage event count (SDPEC) forms a log-normal distribution [30]. In other words, the log-normality of the distribution of SDPEC is the necessary condition that Eq (1) holds. A normal distribution appears under a wide range of situations; if an independent random variable has a finite variance, its sum tends to follow a normal distribution.

The probability distribution of the total count of passage events was examined. We performed a Kolmogorov-Smirnov (KS) test on the null hypothesis that *N*_*t*_ was log-normally distributed. Whether *N*_*t*_ defined as $\sum_{i\in W_{t}}n_{i,t}$ was log-normally distributed, as mentioned in the *Introduction*, and was related to hypothesis (ii). Next, the SDPEC of each ant from each colony was examined. For each ant *i*, the KS test was performed to assess the null hypothesis that *n*_*i*,*t*_ was log-normally distributed (hypothesis (i)).

#### Time series analysis

We examined the stationarities of *n*_*i*,*t*_ and log *n*_*i*,*t*_, as well as their first differences. If stationary, they indicated stochastic processes whose parameters (such as means and variances) changed when shifted in time. Hence, it is appropriate to model the whole investigated period as a simple stochastic process [31]. To evaluate the null hypothesis that *n*_*i*,*t*_ and log *n*_*i*,*t*_ were non-stationary, an augmented Dickey-Fuller (ADF) test was conducted.

Then, the stationarity of the first differences of *n*_*i*,*t*_ and log *n*_*i*,*t*_ were evaluated, respectively. The first differences, *n*_*i*,*t*_ − *n*_*i*,*t*−1_ and log *n*_*i*,*t*_ − log *n*_*i*,*t*−1_, were defined as $\epsilon_{i,t}^{e}$ and *ϵ*_*i*,*t*_, respectively. The ADF test was used to assess if $\epsilon_{i,t}^{e}$ and *ϵ*_*i*,*t*_ were non-stationary. Then, the distribution of *ϵ*_*i*,*t*_ was examined. To assess the null hypothesis that *ϵ*_*i*,*t*_ follows a normal distribution (hypothesis (i)), the KS test was performed on *ϵ*_*i*,*t*_.

Whether *ϵ*_*i*,*t*_ depended on other worker ants in the same colony was also examined (hypothesis (ii)). The Pearson correlation coefficient *ρ*_*i*,*j*_ between *ϵ*_*i*,*t*_ and *ϵ*_*j*,*t*_ was calculated as follows:

$$\begin{array}{c} \rho_{i,j}=\sum_{t\in W_{i,j}}correl\left(\epsilon_{i,t},\epsilon_{j,t}\right), \end{array}$$ (2)

where

$$\begin{array}{c} correl\left(\epsilon_{i,t},\epsilon_{j,t}\right)=\frac{\left(\epsilon_{i,t}-\bar{\epsilon_{i}}\right)\left(\epsilon_{j,t}-\bar{\epsilon_{j}}\right)}{\sqrt{\sum_{t\in W_{i,j}}{\left(\epsilon_{i,t}-\bar{\epsilon_{i}}\right)}^{2}}\sqrt{\sum_{t\in W_{i,j}}{\left(\epsilon_{j,t}-\bar{\epsilon_{j}}\right)}^{2}}}. \end{array}$$ (3)

*W*_*i*,*j*_ is the set of days in which worker ants *i* and *j* are both active. $\bar{\epsilon_{k}}\left(k\in \left\{i,j\right\}\right)$ was defined as follows.

$$\begin{array}{c} \bar{\epsilon_{k}}=\frac{\sum_{t\in W_{i,j}}\epsilon_{k,t}}{\sum_{t\in W_{i,j}}1}. \end{array}$$ (4)

We calculated *ρ*_*i*,*j*_ only for the pairs of days (*i*, *j*) satisfying *T*_*i*,*j*_ ≥ 10, where *T*_*i*,*j*_ is the overlap period between the active period of the ant *i* and that of ant *j* (see Subsection *Data selection* and Fig 2 for the definition of active period).

If Eq (1) holds and *ϵ*_*i*,*t*_ is an independent normal random variable, then *N*_*t*_ does not generally distribute log-normally. Then, there may exist a correlation between *ϵ*_*i*,*t*_’s. Mathematically, hypothesis (ii) was related to this suggestion.

#### Confidence level

Our statical significance level was set at (*p* value) < 0.05 for both static and time series analyses.

## Results

Observation error, selection effect and summary statistics are shown in supporting information. Here, to clearly demonstrate the obtained results, before describing the details, we show our logical flow to examine the hypotheses by identifying the role of each table and figure.

**Fig 3:**
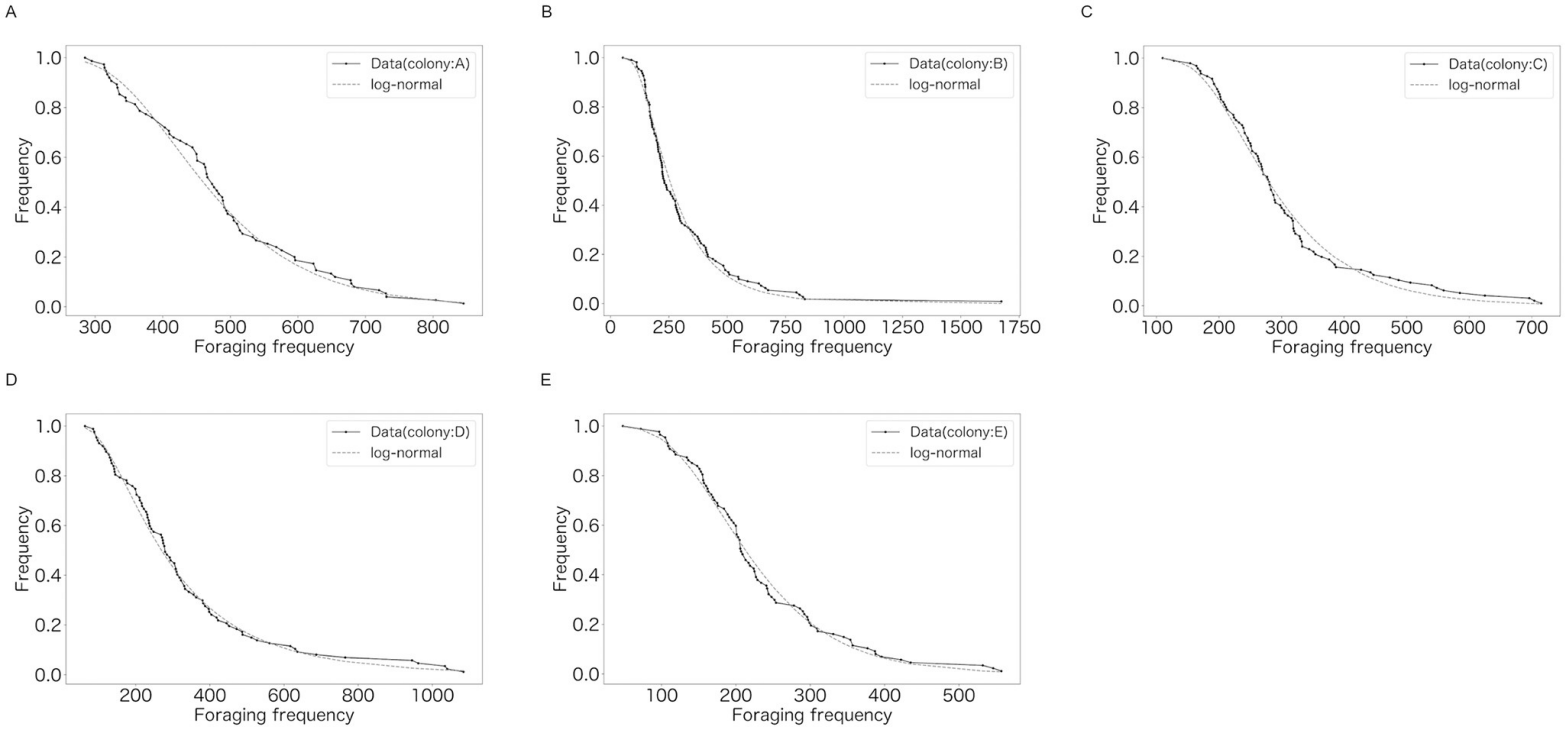
Cumulative probability distributions of *N*_*t*_. The solid lines represent the data. The dashed lines represent the lines of best fit. (A) Colony: A. (B) Colony: B. (C) Colony: C. (D) Colony: D. (E) Colony: E.

**Fig 4:**
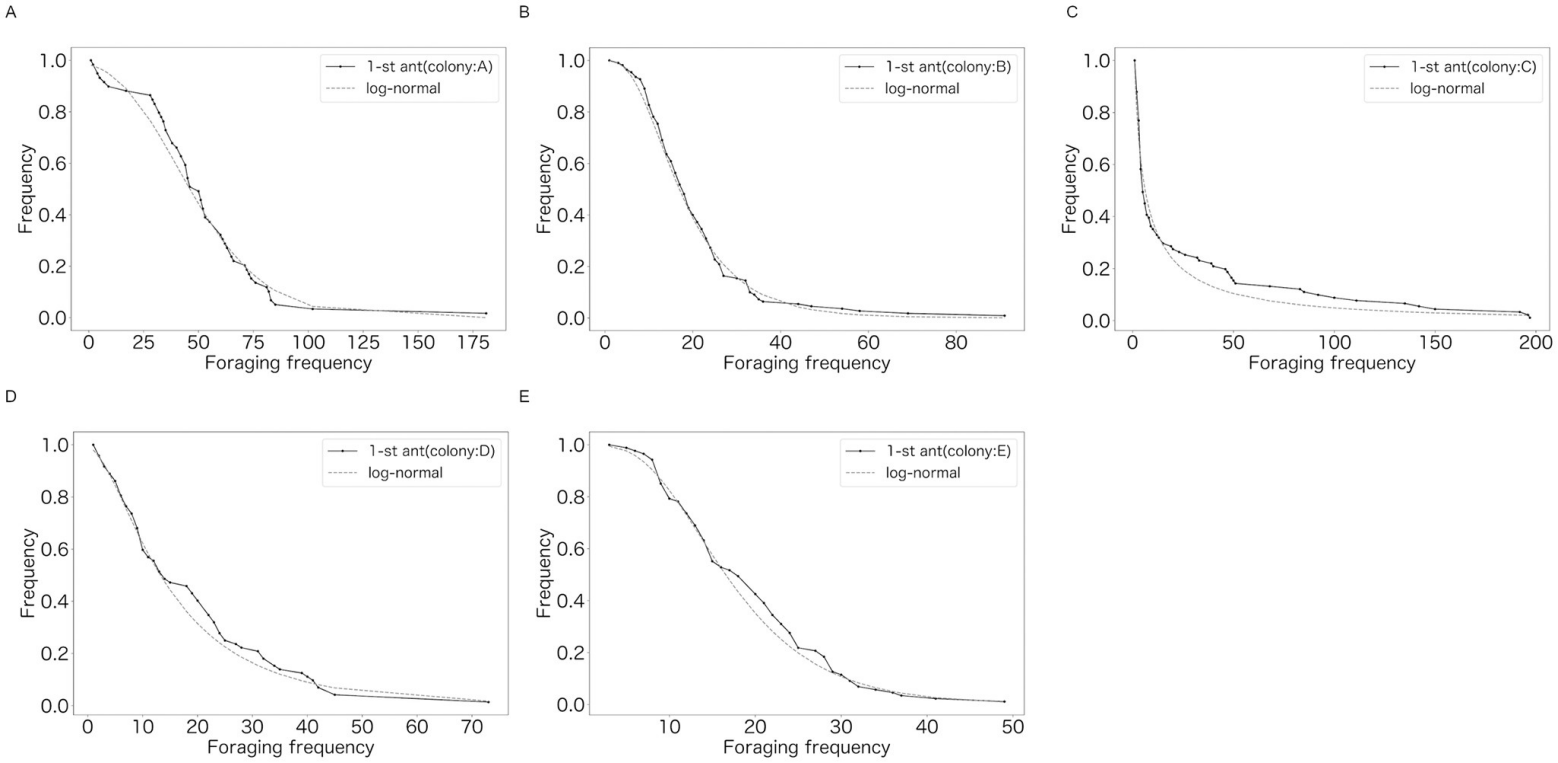
Cumulative probability distributions of *n*_*t*_ for the most active ant in 5 colonies. The solid lines represent the data. The dashed lines represent the lines of best fit. (A) colony: A. (B) colony: B. (C) colony: C. (D) colony: D. (E) colony: E.

**Fig 5:**
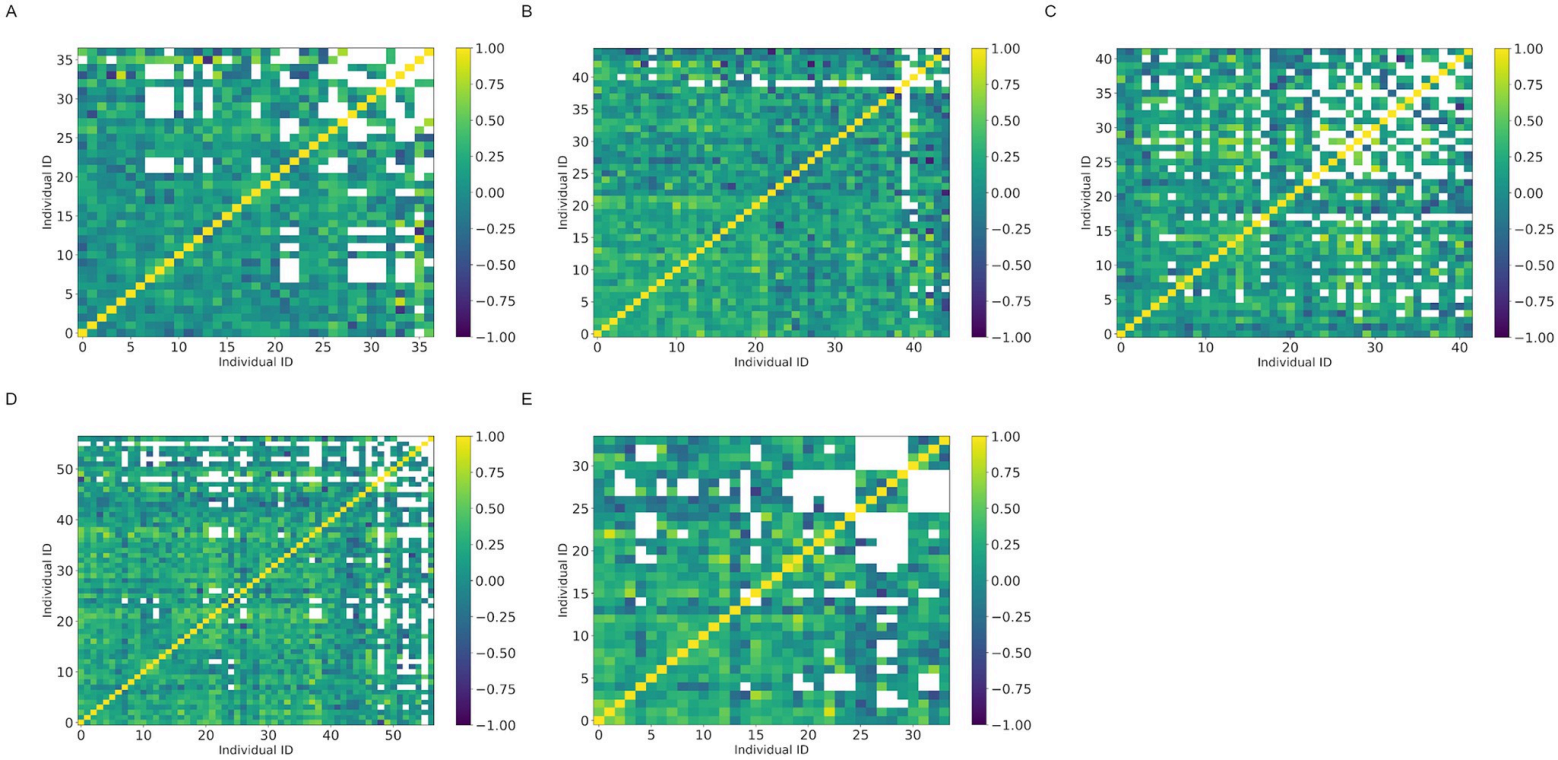
The Pearson correlation coefficient *ρ*_*i*,*j*_ between *ϵ*_*i*,*t*_ and *ϵ*_*j*,*t*_. Vertical and horizontal axes correspond to each worker ant. Colors are differentiated according to the value of *ρ*_*i*,*j*_. White represents *T*_*i*,*j*_ ≤ 10. (A) Colony: A. (B) Colony: B. (C) Colony: C. (D) Colony: D. (E) Colony: E.

**Fig 6:**
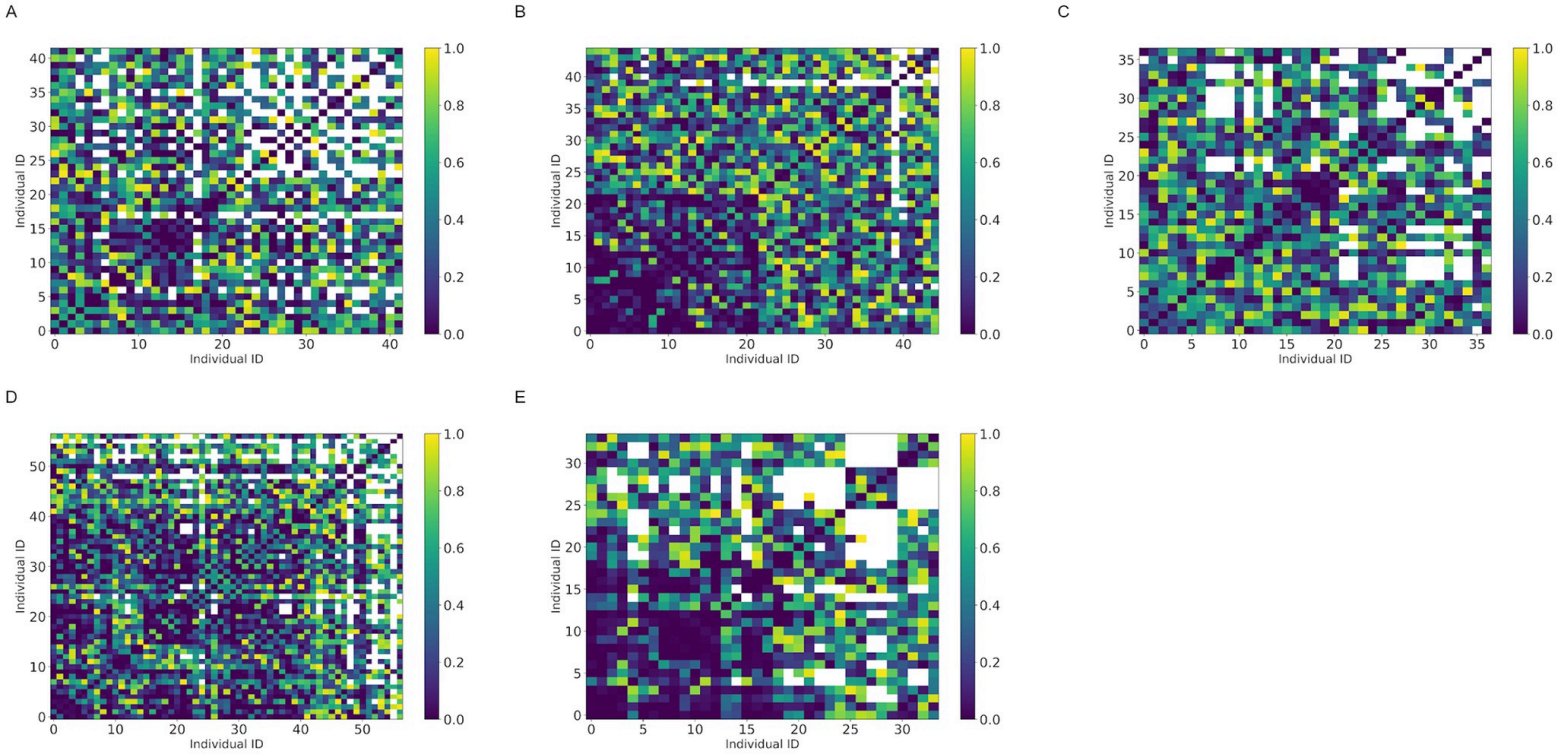
*P* value for the Pearson correlation coefficient. Vertical and horizontal axes correspond to each worker ant. Colors are differentiated according to the *p* value. White represents *T*_*i*,*j*_ ≤ 10. (A) Colony: A. (B) Colony: B. (C) Colony: C. (D) Colony:D. (E) Colony: E.

**Fig 7:**
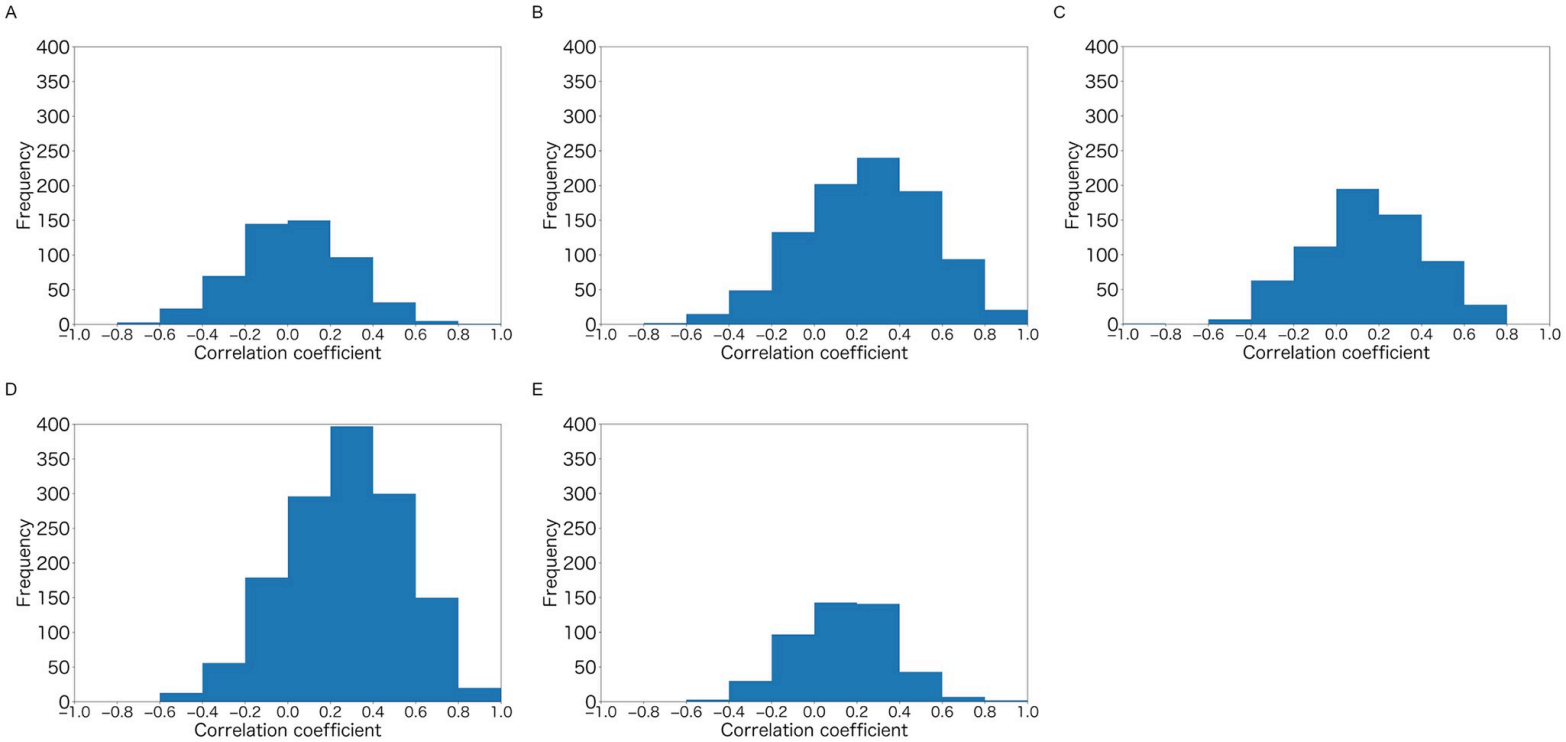
Frequency distribution of the Pearson correlation coefficient *ρ*_*i*,*j*_ between *ϵ*_*i*,*t*_ and *ϵ*_*j*,*t*_. (A) Colony: A. (B) Colony: B. (C) Colony: C. (D) Colony: D. (E) Colony: E.

Fig 1 shows the experimental setups. Fig 2, S1 Fig and Table 1 show images and characteristics of data. S1–S7 Tables show characteristics of the data such as summary statistics. Table 3 and Fig 4 show that the distribution of foraging frequency is consistent with the hypothesis (i). Tables 4 and 5 are the main results that support the hypothesis (i). Next, “random number” in the hypothesis is identified (Table 6). Additionally, Table 2 and Fig 3, together with hypothesis (i), suggest that hypothesis (ii) holds. However, as described in the last part of the introduction, this suggestion based on a mathematical discussion is still not complete. Thus, to complete the logic, we show Figs 5–7 and Table 7 which directly indicate the validity of the hypothesis (ii).

**Table 2:** Results of the Kolmogorov-Smirnov (KS) test for log *N*_*t*_.

Colony	p value
A	0.638086
B	0.348316
C	0.416029
D	0.906069
E	0.871890

### Static analysis

The null hypothesis that *N*_*t*_ was log-normally distributed was not rejected for any colony (Table 2 and Fig 3).

The results of the KS test, to test the null hypothesis that *n*_*i*,*t*_ was log-normally distributed for five colonies, are shown in Table 3. The results for the most active worker ants, showing the largest number of passage events in each colony, are shown in Fig 4. The null hypothesis was not rejected for at least 54.8% of the active worker ants. The proportion of passage events of worker ants that were assumed to have a log-normal distribution was at least 72.4%.

**Table 3:** Results of the Kolmogorov-Smirnov (KS) test for log *n*_*i*,*t*_. The second column shows the sample size for each colony. The third column shows the percentage of samples with p values ≤ 0.05 to the numbers of active worker ants. The fourth column shows the passage counts by samples with p>0.05. The 5th column shows the percentage of passage counts by samples with p>0.05 to passage counts by active worker ants.

Colony	Samples(p>0.05)	Samples (%) (p>0.05)	Passage counts (p>0.05)	Passage counts (%) (p>0.05)
A	23	54.8	26626	73.7
B	38	71.7	27448	81.6
C	38	66.7	21191	72.4
D	47	67.2	24703	85.6
E	31	83.8	18287	91.5

### Time series analysis

The null hypothesis that *n*_*i*,*t*_ was non-stationary was not rejected for more than half of the samples in three colonies (A, C, and E) (Table 4). For stationarity of log *n*_*i*,*t*_, the null hypothesis was not rejected for more than half of the samples in any colony (Table 4). The null hypothesis that $\epsilon_{i,t}^{e}$ and *ϵ*_*i*,*t*_ were non-stationary was rejected for more than 83.8% and 81.1% of the samples, respectively (Table 5). The null hypothesis that *ϵ*_*i*,*t*_ follows a normal distribution was not rejected for at least 73.7% of the samples in each colony (Table 6). For those samples where *ϵ*_*i*,*t*_ can be assumed to have a normal distribution, *n*_*i*,*t*_ can be written as follows:

$$\begin{array}{c} n_{i,t}=n_{i,t-1}\text{exp}\left(\epsilon_{i,t}^{*}\right). \end{array}$$ (5)

where $\epsilon_{i,t}^{*}$ follows normal distribution.

**Table 4:** Results of the augmented Dickey-Fuller (ADF) test for *n*_*i*,*t*_ and log *n*_*i*,*t*_. The second column shows the sample size of each colony. The third and fourth columns show the numbers of samples for which *n*_*i*,*t*_ was stationary at the 5% confidence level and their percentages, respectively. The fifth and sixth columns show the numbers of samples for which log *n*_*i*,*t*_ was stationary at the 5% confidence level and their percentages, respectively.

Colony	Number of samples	*n*_*i*,*t*_	Col3/Col2 (%)	log *n*_*i*,*t*_	Col5/Col2 (%)
A	42	18	42.9	11	26.2
B	53	34	64.2	25	47.2
C	57	14	24.6	8	14.0
D	61	41	67.2	16	26.2
E	37	18	48.6	13	35.1

**Table 5:** Results of the augmented Dickey-Fuller (ADF) test for $\epsilon_{i,t}^{e}$ and *ϵ*_*i*,*t*_. The second column shows the sample size of each colony. The third and fourth columns show the numbers of samples for which $\epsilon_{i,t}^{e}$ was stationary at the 5% confidence level and their percentages, respectively. The fifth and sixth columns show the numbers of samples for which *ϵ*_*i*,*t*_ was stationary at the 5% confidence level and their percentages, respectively.

Colony	Number of samples	$\epsilon_{i,t}^{e}$	Col3/Col2 (%)	*ϵ*_*i*,*t*_	Col5/Col2 (%)
A	42	39	92.9	41	97.6
B	53	46	86.8	47	88,7
C	57	51	89.5	50	87.7
D	61	55	90.2	55	90.2
E	37	31	83.8	30	81.1

**Table 6:** Results of the Kolmogorov–Smirnov(KS) test for normality on $\epsilon_{i,t}^{log}$. Numbers of samples with p values ≥ 0.05 are shown in the third column. The 5th and 6th columns show total passage counts of worker ants in column 2 and column 3, respectively.

Colony	Number of samples	Samples with p≥0.05	Col3/Col2 (%)	Passage counts (col2)	Passage counts (col3)	Col6/Col5 (%)
A	42	37	88.1	36148	34543	95.5
B	53	45	84.9	33623	30156	89.6
C	57	42	73.7	29265	21674	74.0
D	61	57	93.4	28873	28532	98.8
E	37	34	91.9	19982	19065	95.4

The absolute values of *ρ*_*i*,*j*_ were larger than 0.2 for at least 43.9% of the samples in each colony (Fig 5 and Table 7). Correlations were significant (p<0.05) for at least 19.0% of the sample in each colony (Fig 6 and Table 7). We observed *T*_*i*,*j*_ < 10 in 21.0%(colony A), 4.2%(colony B), 23.9%(colony C), 11.6%(colony D), and 16.9%(colony E) of the samples. The frequency distribution of *ρ*_*i*,*j*_ was skewed to the left, and the value of *ρ*_*i*,*j*_ was more likely to be positive, except for colony A (Fig 7).

**Table 7:** Percentage of samples that satisfy |*ρ*_*i*,*j*_| > 0.2(second column), *p* < 0.05(third column), and both conditions(fourth column).

Colony	|*ρ*_*i*,*j*_| > 0.2	*p* < 0.05	|*ρ*_*i*,*j*_| > 0.2 & *p* < 0.05
A	43.9	19.0	19.0
B	64.7	39.3	39.2
C	53.1	23.8	23.8
D	66.3	43.6	43.6
E	48.5	21.5	21.5

## Discussion

For most of the worker ants in this study, SDPEC could be characterized by a log-normal distribution, and the time development of *n*_*j*,*t*_ could be denoted by Eq (5). SDPEC of all the selected worker ants in each colony could be characterized by a log-normal distribution. The results are consistent with hypothesis (i).

The fact that the first differences of *n*_*i*,*t*_ were stationary showed that Eq (5) is valid throughout the active periods. In other words, the daily foraging frequency of the worker ants could be expressed by a single mathematical model throughout their active periods. This suggests the universality of our mathematical model (Eq (5)); the law that expresses the foraging frequency of worker ants does not change regardless of their identity, age, and experience.

As mentioned in section *Introduction* and subsection *time series analysis*, if multiple variables are randomly and independently generated from a log-normal distribution, their sum does not generally follow a log-normal distribution. As both *n*_*i*,*t*_ and *N*_*t*_ followed a log-normal distribution, it indicates that *n*_*i*,*t*_ was not independent of *n*_*j*,*t*_ (*i* ≠ *j*). We found that *ρ*_*i*,*j*_ was substantially different from 0 in some cases. This fact supports hypothesis (ii). The correlation of foraging activities of the worker ants should play a vital role in generating a log-normal distribution of *N*_*t*_. The correlation coefficients *ρ*_*i*,*j*_ were likely to be positive.

The source of the correlation remains unknown. One possible explanation for this is that if a colony needs to increase its food reserves, some worker ants communicate with each other, in the time scale of day or longer, to recognize as a group that they should increase the foraging frequency.

On the shorter timescale, the interaction among workers is essential to generate the periodicity of activity levels of groups of workers [32, 33]. Moreover, the individual movement in the timescale of seconds has universal relation between the velocity and the duration of moving, and the universality is not affected by the social interactions [34].

The relationship between social interactions and the correlation of activity in multiple timescales could generate a long-term correlation. However, certain kinds of social interactions change the temporal characteristics of individual workers [7]. Therefore, it is important to specify what kind of social interaction affects the different timescale correlations in future work.

## Conclusion and future perspective

In this study, we analyzed the foraging activity of ants (*Camponotus japonicus*) in five monodomous colonies. The data consisted of daily counts of the passage events of each ant between a nest chamber and a foraging arena. We developed a successful mathematical model based on time series data of foraging worker ants.

Future research should focus on examining the changes in the probability distribution when the number of worker ants included in the study is intentionally increased or reduced. Given the data, it is possible to answer questions such as 1) whether there is a mechanism to control the activity of worker ants so that SDPEC remains at a log-normal distribution if any worker ants are removed from the colony, or 2) what kind of correlation matrix structure is sufficient to cause the distribution of *N*_*t*_ to be log-normal.

We provided a few explanations to link the experimental results and biological interpretations; another future research avenue is to further examine these links. Since our dataset consists of daily foraging frequency, we could not verify the explanations, therefore a more detailed dataset may prove helpful in this regard.

Moreover, it would be interesting to investigate the advantages of a colony in which SDPEC follows a log-normal distribution. The investigation would possibly suggest that it reflects optimal behavior against risks, such as predation.

## Supporting information

S1 Fig Total count of daily passage events *N*_*t*_. Each horizontal axis represents the duration of each experiment. (A) Colony: A. (B) Colony: B. (C) Colony: C. (D) Colony: D. (E) Colony: E.
(TIF)

S1 Table Number of worker ants in each colony. (TEX)

S2 Table Passage counts for each colony (A-E). (TEX)

S3 Table Summary statistics: Colony A. (TEX)

S4 Table Summary statistics: Colony B. (TEX)

S5 Table Summary statistics: Colony C. (TEX)

S6 Table Summary statistics: Colony D. (TEX)

S7 Table Summary statistics: Colony E. (TEX)
