# Supplementary figures and images for: Characteristics of daily foraging activity of Camponotus japonicus via time series analysis
Source: PLoS One. 2023 Nov 16;18(11):e0293455. doi: 10.1371/journal.pone.0293455 (PMC10653500; doi:10.1371/journal.pone.0293455)

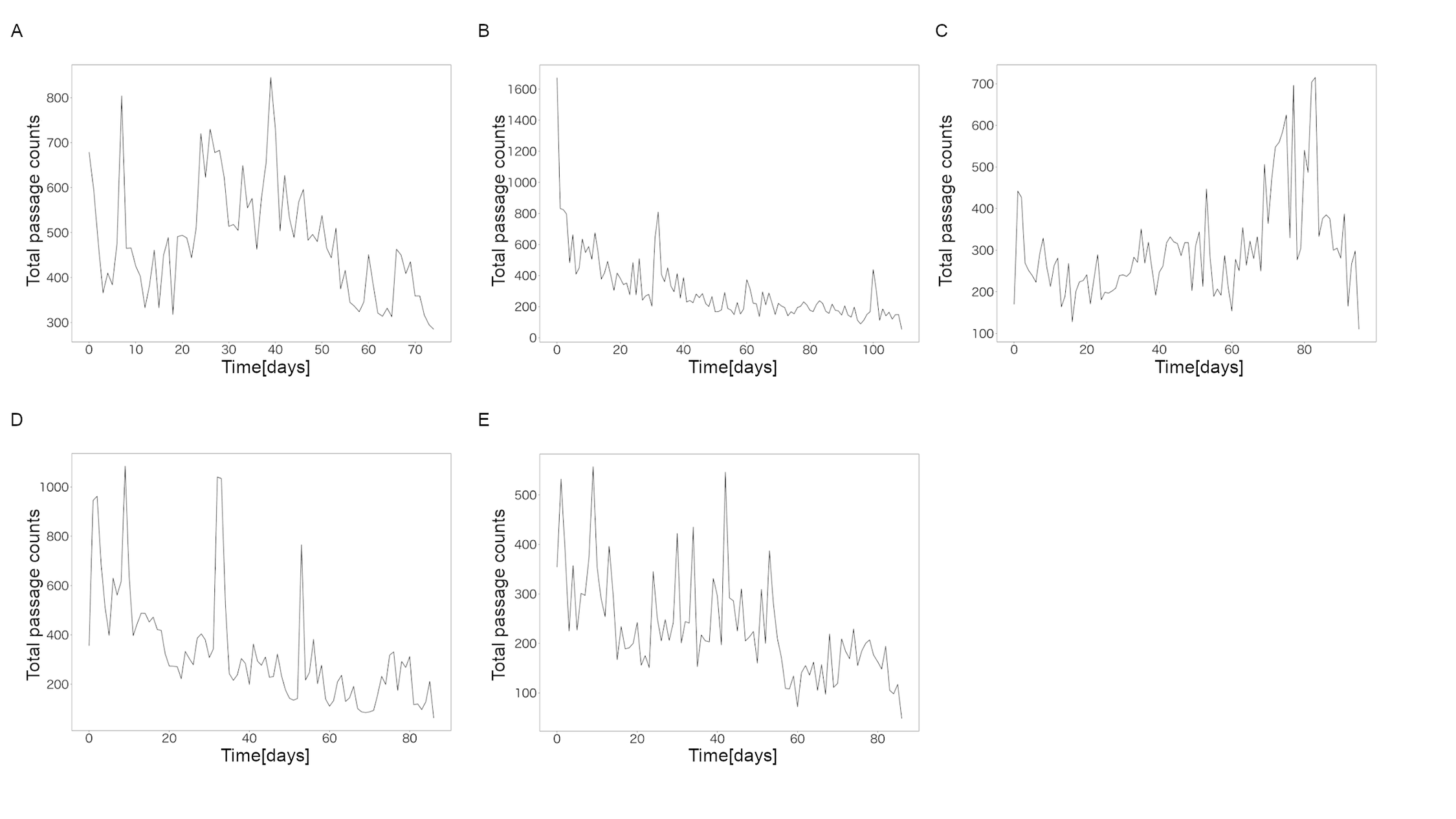

Supplement: S1 Fig — Each horizontal axis represents the duration of each experiment. (A) Colony: A. (B) Colony: B. (C) Colony: C. (D) Colony: D. (E) Colony: E. (TIF) [file pone.0293455.s001.tif]
